# Overall Hemostatic Potential Assay Detects Risk of Progression to Post-Thrombotic Syndrome in Anticoagulated Patients following Deep Vein Thrombosis

**DOI:** 10.3390/diagnostics12123165

**Published:** 2022-12-14

**Authors:** Blake McLeod, Hui Yin Lim, Harshal Nandurkar, Prahlad Ho, Julie Wang

**Affiliations:** 1 Department of Medicine, Northern Health, University of Melbourne, Parkville, Melbourne, VIC 3052, Australia; 2 Department of Hematology, Northern Pathology Victoria, Northern Health, Epping, Melbourne, VIC 3076, Australia; 3 Australian Centre for Blood Diseases, Monash University, Melbourne, VIC 3004, Australia

**Keywords:** post-thrombotic syndrome, overall hemostatic potential, Villalta score, fibrinolysis

## Abstract

Deep vein thrombosis (DVT) frequently leads to post-thrombotic syndrome (PTS) which is challenging to predict and prevent. Identifying those at high risk of developing PTS may help to focus preventative strategies. Adults were recruited within 3 months of DVT diagnosis. Blood was sampled during the therapeutic anticoagulation phase. Overall hemostatic potential (OHP) assay, a spectrophotometric assay, was performed on platelet-poor plasma (PPP). In this assay, fibrin formation is triggered by small amounts of thrombin and termed the overall coagulation potential (OCP). Simultaneously, thrombin and tissue plasminogen activator are added to PPP and the resulting fibrin aggregation curve is the overall hemostatic potential (OHP). Fibrinolysis is expressed by the parameter overall fibrinolytic potential (OFP%). Patients were followed up at regular intervals. PTS was diagnosed if the Villalta score was ≥5 at least 3 months after the DVT diagnosis. Results were obtained from 190 patients (53.7% male, mean age 56.9 years). PTS developed in 62 (32.6%) patients. Patients with PTS displayed significantly higher median OCP (45.8 vs. 38.8 units, *p* = 0.010), OHP (12.8 vs. 9.2 units, *p* = 0.005) and significantly lower OFP (74.1 vs. 75.6%, *p* = 0.050). PTS patients had higher neutrophil/lymphocyte ratios (NLR) (2.3 vs. 1.9, *p* = 0.007). After multivariate analysis, proximal DVT location, history of varicose veins, NLR ≥ 2.6, OHP > 13.0 units and weight >108 kg were independent predictors for PTS. The c-statistic of the multivariate model was 0.77. This pilot study suggests that OHP testing while patients are still anticoagulated may assist in the prediction of PTS development and could assist in prognostication and targeting of preventative measures. However, larger prospective studies are needed to confirm these findings.

## 1. Introduction

Post-thrombotic syndrome (PTS) is a common complication affecting 20–50% of patients following deep venous thrombosis (DVT) [1]. PTS is characterized by chronic symptoms of venous insufficiency that often worsen during the day. Symptoms can vary from mild to severe in up to 5% of affected persons [1], and can range from pain, pruritus, leg heaviness, to chronic edema and venous ulceration [2]. Despite its prevalence, PTS is under-recognized by clinicians and treatment options remain limited. It is associated with significant patient morbidity, reduced productivity and a high cost to the healthcare system [3,4]. PTS patients report reduced quality of life to a degree similar to that of other significant chronic conditions, such as diabetes and heart failure [5,6]. Early identification of patients at high risk of developing PTS may allow for more accurate prognostication and individualized targeting of specific management strategies, such as increased frequency of clinical monitoring to ensure compliance with anticoagulation, graduated compression garments and early referral for endovascular interventions.

The pathophysiology of post-thrombotic syndrome (PTS) remains to be fully elucidated. Current understanding supports a mechanism where thrombosis leads to venous hypertension mediated by a combination of valvular reflux and residual vein obstruction [7,8,9]. Chronic systemic inflammation, vessel wall fibrosis and endothelial dysfunction have also been implicated in this process [10,11]. In line with these hypotheses, PTS has been associated with increased levels of C-reactive protein (CRP) [12,13,14], IL-6 [14], sE-selectin and intracellular adhesion molecule 1 (ICAM-1) [13].

Residual vein obstruction has been identified as a risk factor for PTS [7,9], raising the possibility that hypofibrinolysis is involved by slowing thrombus clearance. Meissner et al. [15] found that tPA and PAI-1 levels at DVT diagnosis were inversely proportional to venous recanalization. Baldwin et al. [16] found that PAI-1 knockout mice had more vein wall fibrosis after DVT. However, evidence from human studies have been contradictory, with no associations found between levels of PAI-1 or tPA and PTS [17,18]. Siudut et al. [17] discovered that PTS is associated with increased TAFI activity, prolonged clot lysis time, and decreased plasma permeability (Ks), indicating a reduced fibrinolytic potential in individuals with PTS.

Global fibrinolytic potential assays may better reflect an individual’s fibrinolytic capability. The overall hemostatic potential (OHP) assay is a global coagulation assay that examines fibrin production and lysis within the same system, which allows for demonstration of the net effect of the interplay within the fibrinolytic system. We had previously demonstrated that OHP assay could be used to risk stratify for VTE recurrence in anticoagulated patients after venous thromboembolism [19]. We hypothesized that the OHP assay could also be used to identify individuals at risk of developing PTS. Our aim in this study was to identify individuals at high risk of PTS, by incorporating OHP assay results tested during anticoagulation following DVT.

## 2. Materials and Methods

### 2.1. Study Participants

Adult patients aged 18 years and older, with VTE diagnosed within 3 months and receiving therapeutic anticoagulation, were recruited from the thrombosis clinic at the Northern Hospital, a tertiary teaching hospital in Victoria, Australia. Only patients with objectively diagnosed lower limb DVT, as confirmed by imaging, were included in this analysis. Exclusion criteria included superficial thrombophlebitis, significant anemia and persons who could not participate in regular reviews or blood tests. Baseline clinical characteristics of patients were collected including age, gender, weight, smoking status, characteristics of the DVT and previous medical history. Proximal DVT was defined as DVT involving the popliteal vein or a more proximal location. The presence of varicose veins was documented based on self-reported history of varicose veins and/or surgery by the time of DVT diagnosis. Written informed consent was acquired from each participant. This study was approved by the Human Research Ethics Committee of Austin Health (Austin429) and Northern Health (HREC14).

### 2.2. Follow Up

Study subjects were followed up at regular timepoints following recruitment. These occurred at least every 3 months during the period of therapeutic anticoagulation, and every 6 to 12 months following anticoagulation cessation, or change to long-term anticoagulation. Visits occurred either in person or over the telephone as necessitated by the COVID-19 pandemic, for up to 3 years following the VTE diagnosis. Additional follow-up occurred at the clinician’s discretion. PTS was diagnosed and the severity was assessed using the Villalta score (see Appendix A Table A1), which was administered at each visit by the treating clinician. The presence of PTS was defined as a Villalta score ≥5 at any point, more than 3 months from the DVT diagnosis. A Villalta score of 5–9 was classified as mild PTS, 10–14 as moderate PTS and ≥15 as severe PTS [20]. During telephone appointments, patients used a self-reported questionnaire adapted from the Villalta score as per the study by Utne et al. [21], which found good agreement with the original Villalta score. A repeat doppler venous ultrasound was performed to assess for residual thrombus burden, prior to cessation of anticoagulation or change to indefinite anticoagulation. A new episode of DVT or PE after the initial Villalta score was defined as recurrent VTE, and this was determined objectively by compression ultrasonography, computed tomography pulmonary angiography (CTPA), or ventilation/perfusion (V/Q) scan. It was defined that superficial thrombophlebitis was not a recurrent VTE.

### 2.3. Laboratory Procedure

Blood was sampled prior to discontinuation of therapeutic anticoagulation by trained phlebotomists via peripheral venipuncture with 21G needle. Standard investigations were conducted using accepted laboratory standards, including full blood count, coagulation studies, D-dimer and renal function tests, thrombophilia screening (including protein C, protein S, antithrombin, factor V Leiden mutation, prothrombin gene mutation), lupus anticoagulant, anticardiolipin IgG, and b2-glycoprotein-1 IgG. The Clauss method was used to measure the fibrinogen levels using the STA^®^ fibrinogen kit. The STA-LIATEST D-Di Plus kit was used to measure D-dimer using the immunoturbidimetric technique. Platelet-poor plasma (PPP) was created by twice centrifuging citrated plasma at 2500 g for 10 min, followed by storage at −80 °C within 2 h of collection. Following thawing at 37 °C, the samples were batch tested for the OHP assay.

### 2.4. Healthy Controls

Using the same methodology as described above, the OHP results of the VTE cohort were compared to those of a previously published cohort of 144 healthy controls (34.7% male, median age 42 years) [22]. The inclusion criteria for healthy controls were strict and included the absence of any known cardiovascular risk factors, a history of thrombosis, use of oral contraceptives, anticoagulants, or antiplatelets, and a negative thrombophilia screen.

### 2.5. Modified Overall Hemostatic Potential (OHP) Assay

**Figure 1:**
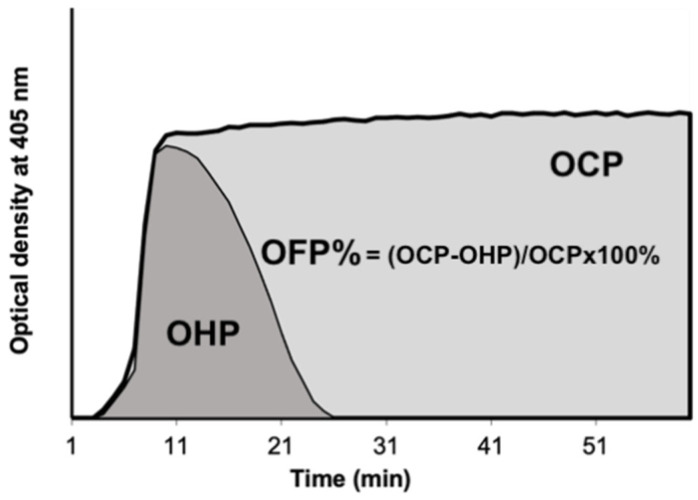
Overall Hemostatic Potential (OHP) assay. The area under the fibrin aggregation curve obtained by the addition of thrombin (0.006 IU/mL) and CaCl_2_ to platelet-poor plasma is the OCP. The area under the fibrin aggregation curve obtained by adding thrombin (0.006 IU/mL), CaCl_2_ and tPA (600 ng/mL) to plasma is the OHP. The OFP% is the difference between the OCP and OHP, expressed as a percentage. Abbreviations: OCP overall coagulation potential; OHP overall hemostatic potential; OFP% overall fibrinolytic potential.

The modified OHP assay is created using a fibrin aggregation curve created by multiple spectrophotometric measurements (Figure 1). Our approach was modified from Curnow et al.’s [23] work. Wells contained 75 μL of thawed PPP to which 75 μL of buffer containing either (i) Tris, NaCl, CaCl2 (final concentration 66 nM Tris, 130 mM NaCl, 35 mM CaCl2; pH 7.0) and thrombin (0.006 IU/mL) to generate the overall coagulation potential (OCP) or (ii) Tris, NaCl, CaCl2, thrombin and tissue plasminogen activator (tPA) (600 ng/mL) to generate the overall hemostatic potential (OHP). The FLUOstar Optima (BMG Labtech, Ortenberg, Germany) plate reader at 405 nm is used to derive the two fibrin–aggregation curves (OCP and OHP) cumulatively. The overall fibrinolytic potential (OFP%) is calculated as the difference between the area under the two curves.

### 2.6. Statistical Analysis

Stata version 17.0 was used to conduct the statistical analysis (StataCorp, College Station, TX, USA). Student’s *t*-tests were used to compare patient groups for the variables that had a normal distribution, and were shown as means and standard deviation. For the variables that were discovered to have a non-normal distribution, Mann–Whitney (rank-sum) tests were used, and these variables were displayed as medians and interquartile ranges. The Shapiro–Wilks test was used to determine normality of variables. Chi-squared tests were used to look for differences in categorical variables that were presented as counts and frequencies. To account for confounders, such as weight, in a multivariate analysis, skewed variables were first transformed into a normal distribution before a linear regression was run. Statistical significance was defined as a two-tailed *p*-value of 0.05. Using the variables determined to be statistically significant in Table 1 and Table 2, a multivariate predictive model was created with the presence of PTS as the endpoint. To find potential multivariate final candidate models, logistic regression was used. Comparing model fit and choosing the best model involved using C-statistics (area under the receiver operating curve), Schwarz’ Bayesian Information Criterion, Akaike Information Criterion and the Hosmer–Lemeshow test.

## 3. Results

**Figure 2:**
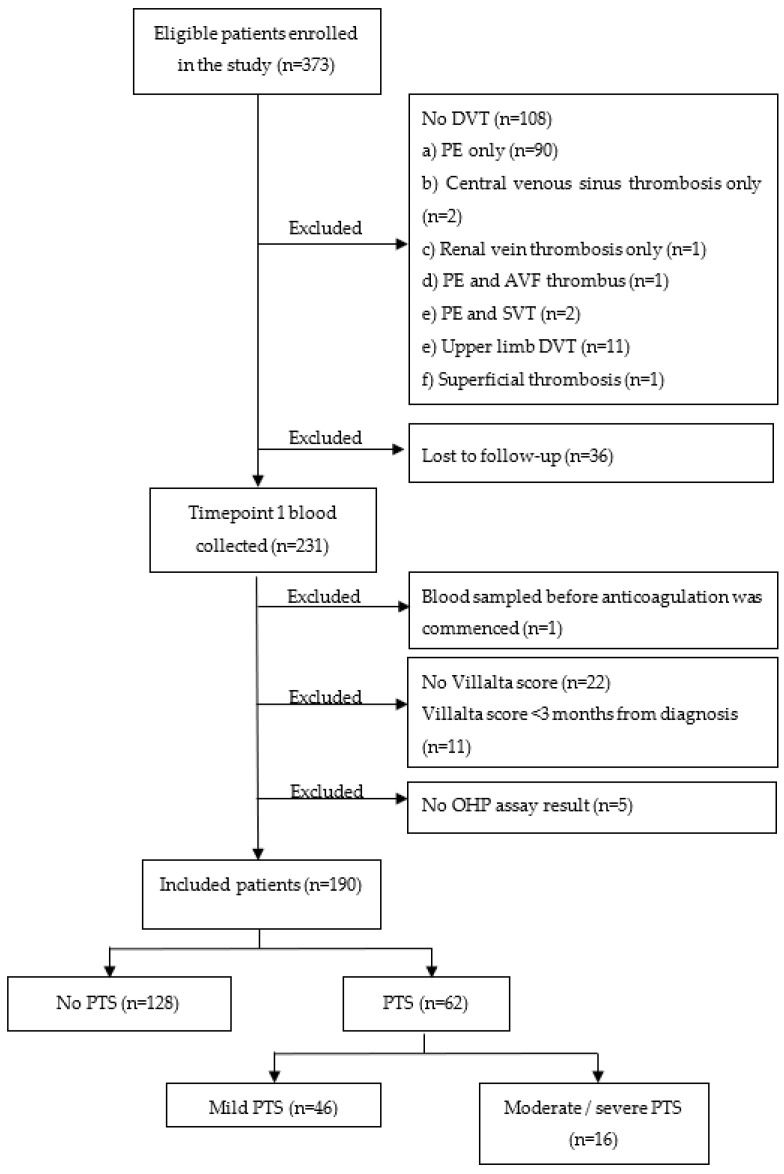
Consort diagram illustrating the inclusion and exclusion of study participants. Abbreviations: PTS, post-thrombotic syndrome; DVT, deep vein thrombosis; PE, pulmonary embolus; AVF, aorto-venous fistula; SVT, superficial venous thrombosis; OHP, overall hemostatic potential.

Between 1 February 2018 and 1 July 2022, 373 patients were recruited into the study. Figure 2 displays study participants in a consort diagram. Excluded patients included 108 without DVT, 36 patients lost to follow-up, 22 without Villalta scores, 5 without OHP assay results, 11 with Villalta scores performed <3 months from DVT diagnosis and 1 patient whose blood was sampled before anticoagulation was commenced. One-hundred and ninety patients remained in the final analysis, of which 102 (53.7%) were male with a mean age of 56.9 years (SD 13.3). The median follow up was 643.5 days [IQR 270.0, 1085.0] Anticoagulation comprised warfarin in 28 (14.7%), direct oral anticoagulants (DOACs) in 156 (82.1%) and enoxaparin in 6 (3.2%) patients. Patients anticoagulated with warfarin weighed significantly more than non-warfarinized patients (128 kg vs. 93 kg, *p* < 0.001). PTS was diagnosed in 62 (32.6%) patients, of which 46 (24.2%) had mild PTS (Villalta score 5–9) and 17 (8.9%) had moderate/severe symptoms (Villalta score ≥ 10).

Table 1 displays the clinical characteristics of patients according to the presence and severity of PTS. Patient with PTS were more likely to demonstrate increased weight (100.0 vs. 92.0 kg, *p* = 0.016), be anticoagulated with warfarin (22.6% vs. 10.9%, *p* = 0.034), have proximal DVT (62.9% vs. 43.8%, *p* = 0.013) and a history of varicose veins (22.6 vs. 4.7%, *p* < 0.001). There were no significant differences between groups with regards to age, sex, unprovoked DVT, previous history of DVT, family history of DVT, smoking, residual thrombus on repeat ultrasound, thrombophilia status or presence of antiphospholipid syndrome. The rates of recurrent VTE (including pulmonary embolus (PE) and/or DVT) or recurrent ipsilateral DVT were comparable between groups. The clinical characteristics were not predictive of the severity of PTS.

**Table 1:** Clinical characteristics of patients with and without post-thrombotic syndrome. Data are *n* (%), and median [interquartile range, IQR] unless specified otherwise.

	No PTS	PTS	*p*-Value *	Mild PTS	Moderate/ Severe PTS	*p*-Value **
*n*	128	62		46	16	
Age (years), mean (SD)	56.6 (14.2)	57.4 (11.2)	0.72	58.2 (11.1)	54.9 (11.6)	0.32
Male	72 (56.2%)	30 (48.4%)	0.31	19 (41.3%)	11 (68.8%)	0.06
Female	56 (43.8%)	32 (51.6%)		27 (58.7%)	5 (31.2%)	
Weight (kg)	92.0 [81.0, 104.0]	100.0 [85.0, 120.0]	**0.016**	97.0 [80.0, 116.0]	110.0 [88.5, 129.0]	0.07
Unprovoked DVT	77 (60.2%)	43 (69.4%)	0.22	30 (65.2%)	13 (81.2%)	0.23
Proximal DVT	56 (43.8%)	39 (62.9%)	**0.013**	27 (58.7%)	12 (75.0%)	0.24
Previous DVT history	32 (25.0%)	14 (22.6%)	0.72	12 (26.1%)	2 (12.5%)	0.26
Family history DVT/PE	22 (17.2%)	15 (24.2%)	0.25	12 (26.1%)	3 (18.8%)	0.56
Malignancy	10 (7.8%)	2 (3.2%)	0.22	2 (4.3%)	0 (0.0%)	0.40
Smoker	17 (13.6%)	13 (21.0%)	0.20	11 (23.9%)	2 (12.5%)	0.33
History of varicose veins	6 (4.7%)	14 (22.6%)	**<0.001**	11 (23.9%)	3 (18.8%)	0.67
Length of follow up (days)	480.5 [261.0, 1072.0]	815.0 [368.0, 1127.0]	0.07	820.5 [357.0, 1119.0]	761.0 [405.5, 1127.5]	1.00
Anticoagulation						
DOAC	110 (85.9%)	46 (74.2)	**0.048**	37 (80.4%)	9 (56.2%)	0.06
Warfarin	14 (10.9%)	14 (22.6%)	**0.034**	8 (17.4%)	6 (37.5%)	0.10
Enoxaparin	4 (3.1%)	2 (3.2%)	0.97	1 (2.2%)	1 (6.2%)	0.43
Residual thrombus on repeat imaging	50 (42.4%)	26 (43.3%)	0.90	18 (40.9%)	8 (50.0%)	0.53
Inherited thrombophilia #	25 (19.7%)	10 (16.4%)	0.59	7 (15.6%)	3 (18.8%)	0.77
Antiphospholipid syndrome ^	3 (2.4%)	1 (1.6%)	0.75	1 (2.2%)	0 (0.0%)	0.55
Recurrent VTE during follow-up	12 (9.4%)	6 (9.7%)	0.95	4 (8.7%)	2 (12.5%)	0.66
Recurrent ipsilateral leg DVT during follow-up	5 (3.9%)	4 (6.5%)	0.44	3 (6.5%)	1 (6.2%)	0.97

Abbreviations: PTS, post-thrombotic syndrome; SD, standard deviation; DVT, deep vein thrombosis; VTE, venous thromboembolism; PE, pulmonary embolus; DOAC, direct oral anticoagulant; *p*-values in bold are <0.05 * No PTS vs. PTS ** Mild PTS vs. moderate/severe PTS; # Inherited thrombophilia was defined by factor V Leiden mutation, prothrombin gene mutation, protein C deficiency, protein S deficiency or antithrombin deficiency; ^ antiphospholipid syndrome was identified using the Sydney criteria [24].

Table 2 displays baseline characteristics and OHP results for 144 healthy controls and DVT patients according to PTS status. After adjusting for age and sex, DVT patients showed significantly higher OCP and OHP and significantly lower OFP% compared to healthy controls, regardless of PTS status.

**Table 2:** OHP results and baseline characteristics for healthy controls and DVT patients, according to PTS status. Data are *n* (%), and median [interquartile range, IQR] unless specified otherwise.

	Healthy Controls (HC)	No PTS	PTS	*p*-Value HC vs. No PTS	*p*-Value HC vs. PTS
*n*	144	128	62		
Age (years)	42.0 [24.5, 57.0]	57.0 [46.0, 67.0]	58.5 [51.0, 65.0]	**<0.001**	**<0.001**
Male	50 (34.7%)	72 (56.2%)	30 (48.4%)	**<0.001**	**0.065**
Fibrinogen (g/L)	2.9 [2.5, 3.5]	3.5 [3.0, 4.2]	3.9 [3.4, 4.6]	**<0.001**	**<0.001**
D-dimer (mg/L FEU)	0.2 [0.1, 0.3]	0.3 [0.3, 0.5]	0.3 [0.3, 0.5]	**<0.001**	**<0.001**
Factor VIII (%)	106.0 [86.0, 145.0]	148.0 [112.5, 190.5]	162.0 [121.5, 202.5]	**<0.001**	**<0.001**
von-Willebrand antigen (%)	102.0 [87.0, 142.5]	148.5 [101.0, 192.0]	159.5 [118.5, 200.0]	**0.005**	**<0.001**
OCP (units)	34.5 [29.0, 43.3]	38.8 [32.1, 48.1]	45.8 [39.3, 52.8]	**0.004**	**<0.001**
OHP (units)	6.4 [4.8, 9.5]	9.2 [6.9, 13.2]	12.8 [8.8, 17.2]	**<0.001**	**<0.001**
OFP (%)	81.1 [77.5, 84.1]	75.6 [71.0, 80.5]	74.1 [64.9, 77.3]	**<0.001**	**<0.001**

Abbreviations: DVT, deep vein thrombosis; OCP, overall coagulation potential; OHP, overall hemostatic potential; OFP, overall fibrinolytic potential; All *p*-values are adjusted for age and gender, boldened values signify *p* < 0.05.

When compared with patients without PTS, and adjusted for the confounding effect of weight, patients with PTS showed significantly higher median OCP (45.8 vs. 38.8 units, *p* = 0.010), OHP (12.8 vs. 9.2 units, *p* = 0.005) and significantly lower OFP (74.1 vs. 75.6%, *p* = 0.050) (Table 3). There were no significant differences in OCP, OHP and OFP between mild and moderate/severe PTS. The presence of PTS was also associated with significantly lower lymphocytes (1.8 vs. 2.2 × 10^9^/L, *p* = 0.007) and higher neutrophil/lymphocyte ratio (NLR) (2.3 vs. 1.9, *p* = 0.007). Median fibrinogen was elevated in patients with PTS, but after adjusting for weight this became not statistically significant (3.9 vs. 3.5 g/L, *p* = 0.06). D-dimer, factor VIII and von-Willebrand antigen showed no differences between groups. Following multivariate logistic regression (Table 4), the factors independently associated with increased risk of developing PTS were proximal location of DVT, history of varicose veins, NLR ≥ 2.6 (highest quartile), OHP > 13.0 units (highest tertile) and weight >108 kg (highest quartile). The c-statistic of the multivariate model was 0.77. 

## 4. Discussion

The fibrinolytic system has long been postulated to be a critical contributor to the development of chronic thrombotic complications such as post-thrombotic syndrome. To the best of our knowledge, this pilot study is the first to utilize the OHP assay to identify persons diagnosed with DVT at risk of developing PTS. These pilot results show that significantly higher fibrin generation potential and reduced fibrinolytic potential were detected in patients who subsequently developed PTS, with OHP in the highest tertile (>13.0 units) being an independent predictor of increased risk of PTS. These changes were detected despite patients still being anticoagulated at the time of blood sampling. The ability to test during anticoagulation is a major strength, as it allows prediction of PTS early in the treatment course to enable more effective and timely interventions to be adopted.

Previous biomarker studies in PTS have shown significant heterogeneity and conflicting results. Evidence on the association of D-dimer with PTS is conflicting, which may be due to differences in blood sampling timing, measurement method and method of PTS diagnosis [25]. We found no correlation between D-dimer and the presence or severity of PTS, which is consistent with other studies in which D-dimer was tested during anticoagulation [13,26]. This may be attributable to the observation that D-dimer is suppressed by anticoagulation [19]. Unlike other studies [9,27], we found no link between residual venous obstruction and the development of PTS. However, the timing of repeat imaging was at the discretion of the clinician, and was performed by different operators without the routine use of standardized criteria to assess residual thrombus (such as the Prandoni score). Nonetheless, the methods in this study are reflective of real world VTE management where residual thrombus reporting remains highly heterogenous.

Recently, there has been much interest in exploring the relationship between inflammatory biomarkers and cardiovascular disease. Neutrophil/lymphocyte ratio (NLR) and platelet/lymphocyte ratio (PLR) are biomarkers that may reflect the balance between inflammation and the adaptive immune system, and have been associated with increased risk of thromboembolic disease and all-cause mortality [28,29,30,31]. Higher pre-operative NLR and PLR were independent predictors of DVT complicating total knee arthroplasty [32]. NLR at DVT diagnosis has been shown to correlate with higher thrombus burden and more proximal location [33]. NLR > 95th percentile was associated with a 2.4 times higher risk of developing VTE in the Tromso study [34]. Our finding that NLR in the highest quartile (>2.6) was associated with the development of PTS, is in keeping with the suggestion that inflammation is important to the pathophysiology of PTS [12,13]. While CRP results were not available in this study, other acute phase reactants, such as Factor VIII and von-Willebrand antigen levels, were comparable between patients with PTS and those without. PTS patients also did not show significantly higher fibrinogen levels, similar to previous studies [17,18,25]. 

Consistent with previous studies, increased weight, proximal location of DVT and a history of varicose veins were significantly and independently associated with the development of PTS in the multivariate analysis [27]. We also found that a higher proportion of patients with PTS were on warfarin. A 2021 systematic review [35] found that patients anticoagulated with DOACs were significantly less likely to develop PTS than those treated with warfarin (odds ratio 0.52, *p* < 0.001). The mechanism for this finding may be due to DOACs having more stable pharmacokinetics and possessing anti-inflammatory properties [35,36]. In those treated with warfarin following DVT, previous research has shown an increased incidence of PTS in those who spent >50% of time with INR below 2.0 [37], indicating that the quality of anticoagulation may directly affect PTS development. However, patients in our study who were warfarinized weighed significantly more than those who were anticoagulated with other agents and obesity may be a potential confounder. This may explain why the use of warfarin lost statistical significance as a predictor of PTS in the multivariate analysis.

While clinical risk factors for PTS are well established, predicting the risk at an individual level remains difficult. Rabinovich et al. [38] derived the SOX-PTS score from 722 patients which included three independent predictors: BMI > 35 kg/m^2^, DVT in the iliac vein and moderate-severe Villalta severity at DVT diagnosis. However, the c-statistic for this score was only 0.65 and it has not been externally validated. Integrating biomarkers may improve the precision of risk prediction tools on an individual level. Our model, which includes two biomarkers (NLR and OHP) and three clinical factors, has high discriminatory power and could be adapted to a clinical risk prediction score.

There are several limitations to our pilot study, including inconsistent follow-up times and a relatively small sample size. In these interim results, only six patients were classified as having severe PTS; hence, the moderate and severe groups had to be combined in the analysis. Due to the occurrence of the COVID-19 pandemic, some follow ups were missed, delayed, or inconsistently spaced. Furthermore, there was a trend toward longer length of follow up for PTS patients (815.0 vs. 480.5 days, *p* = 0.07). As PTS can develop from 3 to 6 months to 2 years following a DVT [1], it is possible that some individuals in the group without PTS had not yet developed PTS. Additionally, despite every effort being made to collect data prospectively, some data had to be collected retrospectively via medical records and consequently some data may be incomplete. Nevertheless, to our knowledge, this is the first study to investigate the use of global coagulation assay such as OHP to predict an individual’s risk of PTS.

## 5. Conclusions

This pilot study found that the testing of OHP assay in patients receiving anticoagulation was able to detect significantly higher fibrin generation and lower fibrinolytic potential in patients who subsequently developed PTS. In a multivariate model, OHP in the highest quartile was found to be an independent predictor of PTS. Other predictors included increased weight, proximal location of DVT, history of varicose veins and high NLR. Our novel findings highlight the need for larger prospective studies to incorporate biomarkers in improving the prognostication and prevention of PTS. 

## Figures and Tables

**Table 3:** Results of biomarkers according to post-thrombotic syndrome. Data are in *n* (%), and median [interquartile range, IQR].

	No PTS	PTS	*p*-Value *	Mild PTS	Moderate/Severe PTS	*p*-Value **
*n*	128	62		46	16	
Days from DVT diagnosis to blood sample	90.0 [46.0, 112.0]	80.0 [55.0, 105.0]	0.76	82.0 [63.0, 105.0]	67.0 [50.0, 108.0]	0.38
Hemoglobin (g/L)	146.0 [136.0, 155.0]	144.0 [135.0, 154.0]	0.44	140.0 [135.0, 154.0]	148.5 [140.0, 154.5]	0.25
Neutrophils (×10^9^/L)	3.9 (3.3, 5.2]	4.4 [3.5, 5.4]	0.23	4.2 [3.5, 5.4]	4.8 [3.9, 5.8]	0.25
Lymphocytes (×10^9^/L)	2.2 [1.8, 2.8]	1.8 [1.5, 2.3]	**0.007**	1.8 [1.6, 2.3]	1.7 [1.4, 2.3]	0.65
Neutrophil/lymphocyte ratio	1.9 [1.4, 2.4]	2.3 [1.6, 3.0]	**0.007**	2.2 [1.5, 3.0]	2.3 [1.8, 3.9]	0.55
Platelets (×10^9^/L)	256.0 [226.0, 316.5]	259.0 [217.0, 311.0]	0.66	255.0 [231.0, 296.0]	272.0 [214.5, 337.5]	0.48
Platelet/lymphocyte ratio	120.5 [96.3, 148.4]	141.8 [110.0, 184.2]	0.07	138.7 [109.5, 164.4]	150.1 [114.5, 201.6]	0.43
Fibrinogen (g/L)	3.5 [3.0, 4.2]	3.9 [3.4, 4.6]	0.06	3.7 [3.4, 4.6]	4.3 [3.8, 5.0]	0.08
Fibrinogen/lymphocyte ratio	1.6 [1.2, 2.1]	1.9 [1.7, 2.8]	0.001	1.9 [1.6, 2.7]	2.3 [1.7, 3.7]	0.47
D-dimer (mg/L FEU)	0.3 [0.3, 0.5]	0.3 [0.3, 0.5]	0.73	0.3 [0.3, 0.4]	0.4 [0.3, 0.7]	0.33
Factor VIII (%)	148.0 [112.5, 190.5]	162.0 [121.5, 202.5]	0.21	158.0 [128.0, 198.0]	166.0 [113.0, 214.0]	0.76
von-Willebrand antigen (%)	148.5 (101.0, 192.0]	159.5 (118.5, 200.0]	0.51	162.0 (122.0, 200.0]	147.0 (100.0, 200.0]	0.56
OCP (units)	38.8 [32.1, 48.1]	45.8 [39.3, 52.8]	**0.010**	44.3 [40.5, 55.8]	49.3 [32.8, 52.5]	0.71
OHP (units)	9.2 [6.9, 13.2]	12.8 [8.8, 17.2]	**0.005**	12.3 [8.8, 16.8]	13.7 [9.2, 17.2]	0.61
OFP (%)	75.6 [71.0, 80.5]	74.1 [64.9, 77.3]	**0.050**	75.2 [64.9, 77.3]	72.7 [65.7, 76.9]	0.46

Abbreviations: PTS, post-thrombotic syndrome; DVT, deep vein thrombosis; OCP, overall coagulation potential; OHP, overall hemostatic potential; OFP, overall fibrinolytic potential. * *p*-values compare no PTS and PTS, adjusted for weight; ** compares mild PTS and moderate/severe PTS; boldened values signify *p* < 0.05.

**Table 4:** Multivariate logistic model for prediction of developing post-thrombotic syndrome.

	Odds Ratio	Standard Error	*p*-Value	95% Confidence Interval of Odds Ratio
Proximal DVT	2.22	0.79	**0.026**	1.10–4.47
History of varicose veins	7.51	4.28	**<0.001**	2.46–22.96
NLR ≥ 2.6	2.35	0.95	**0.035**	1.06–5.18
OHP > 13.0 units	2.17	0.79	**0.033**	1.06–4.43
Weight > 108 kg	2.86	1.11	**0.007**	1.34–6.11

Abbreviations: DVT, deep vein thrombosis; NLR, neutrophil/lymphocyte ratio; OHP, overall hemostatic potential; boldened values signify *p* < 0.05.

## Data Availability

Not applicable.

## Appendix A

**Table A1:** Villalta score.

Symptoms and Signs	None	Mild	Moderate	Severe
Symptoms				
Pain	0	1	2	3
Cramps	0	1	2	3
Heaviness	0	1	2	3
Paresthesia	0	1	2	3
Pruritus	0	1	2	3
Clinical signs				
Pretibial edema	0	1	2	3
Skin induration	0	1	2	3
Hyperpigmentation	0	1	2	3
Redness	0	1	2	3
Venous ectasia	0	1	2	3
Pain on calf compression	0	1	2	3
Venous ulceration	Absent			Present

Mild PTS: 5–9; Moderate PTS: 10–14; Severe PTS: ≥15; presence of venous ulcer = score 15.
